# A Data-Driven Approach to SAR Data-Focusing

**DOI:** 10.3390/s19071649

**Published:** 2019-04-06

**Authors:** Cataldo Guaragnella, Tiziana D’Orazio

**Affiliations:** 1 DEI—Department of Electrical and Information Engineering, Politecnico di Bari, 70126 Bari, Italy; 2 STIIMA—Institute of Intelligent Industrial Technologies and Systems for Advanced Manufacturing, CNR—Italian National Research Council, 70124 Bari, Italy; tizianarita.dorazio@cnr.it

**Keywords:** SAR system, efficient focusing of SAR data, inverse problem, remote sensing, SAR data-focusing, synthetic aperture radar, Singular Value Decomposition, blind deconvolution, signal processing, parameter estimation, computational modeling

## Abstract

Synthetic Aperture RADAR (SAR) is a radar imaging technique in which the relative motion of the sensor is used to synthesize a very long antenna and obtain high spatial resolution. Several algorithms for SAR data-focusing are well established and used by space agencies. Such algorithms are model-based, i.e., the radiometric and geometric information about the specific sensor must be well known, together with the ancillary data information acquired on board the platform. In the development of low-cost and lightweight SAR sensors, to be used in several application fields, the precise mission parameters and the knowledge of all the specific geometric and radiometric information about the sensor might complicate the hardware and software requirements. Despite SAR data processing being a well-established imaging technique, the proposed algorithm aims to exploit the SAR coherent illumination, demonstrating the possibility of extracting the reference functions, both in range and azimuth directions, when a strong point scatterer (either natural or manmade) is present in the scene. The Singular Value Decomposition is used to exploit the inherent redundancy present in the raw data matrix, and phase unwrapping and polynomial fitting are used to reconstruct clean versions of the reference functions. Fairly focused images on both synthetic and real raw data matrices without the knowledge of mission parameters and ancillary data information can be obtained; as a byproduct, azimuth beam pattern and estimates of a few other parameters have been extracted from the raw data itself. In a previous paper, authors introduced a preliminary work dealing with this problem and able to obtain good-quality images, if compared to the standard processing techniques. In this work, the proposed technique is described, and performance parameters are extracted to compare the proposed approach to RD, showing good adherence of the focused images and pulse responses.

## 1. Introduction

The Synthetic Aperture Radar (SAR) [1,2,3,4] can acquire very-high-resolution images of the inspected area using high bandwidth of the transmitted coherent illumination signal by means of an accurate processing of the ground-received returns. In a standard structure, the system is composed of a platform (i.e., airborne or satellite) using the same antenna both for the transmitting and receiving phases; the target scene is repeatedly illuminated with pulses of radio waves. The signal echoes are emitted at equispaced positions along the satellite track, and their returns are received in the band of the transmitted pulse, converted, and IQ-sampled to produce the baseband complex raw data. A baseband algorithm implements the synthetic aperture to produce the equivalent return of a very narrow azimuth antenna beam. Three main algorithms are available to obtain such high-quality images, namely Range Doppler [5] algorithm, Ω-K [6] algorithm and Chirp Scaling algorithm [7]. All such algorithms, which perform equally in terms of focused image quality [5], require precise geometric acquisition parameters and radiometric parameters. Such parameters are always available as a side documentation of each acquired image.

**Figure 1:**
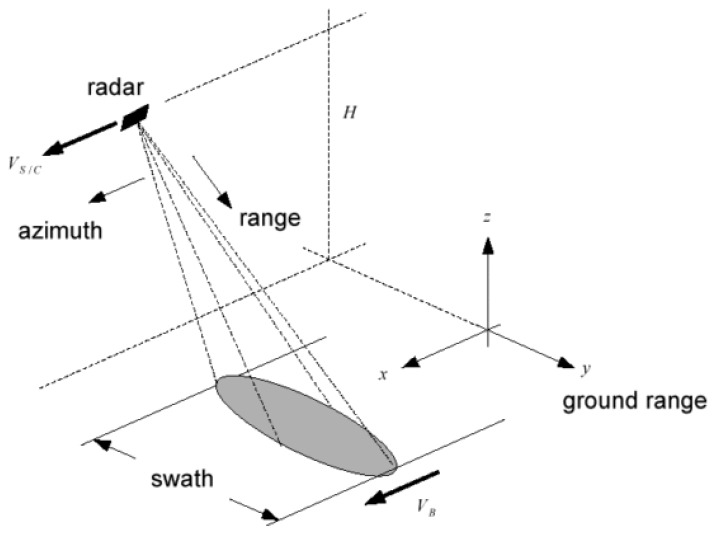
SAR Data acquisition geometry.

Figure 1 reports the acquisition geometry of a SAR system. The synthesis procedure in focusing the acquired data is carried out by coherent integration. Each target on the ground contributes to the radar return on several subsequent transmitted pulses. In SAR two main directions are important to focus the data: slant range direction, in which transmitted pulses travel, and azimuth direction, i.e., the direction of sensor movement. The precise knowledge of the geometry of the acquisition allows to add in phase all the contributes of each single point scatterer on the ground present in the data to obtain the focused image. The wider the beam, the smaller the detail acquired by any return, but the larger the integration size of the track contributions to synthesize the image. The practical azimuth resolution is limited by the PRF choice (the Pulse Repetition Frequency of transmitted pulses used for coherent illumination of the target area), i.e., the azimuth sampling frequency.

In several cases, due to imprecise knowledge of the satellite or aerial vehicle acquisition geometry or due to the presence of motion in the scene (ships, cars, etc.) a bad quality of the image is obtained (defocusing); many authors addressed the problem of a post processing procedure able to exploit the residual correlation present in data to perform accurate focusing of the image. Motion compensation is very important to achieve high resolution in SAR imagery. The phase errors that may be present on the focused image can be compensated through Inertial Measurement Unit (IMU) and Global Positioning System (GPS) side information. However, the need to measure and add such information to the ancillary data complicates the burden on any motion compensation system. In this cases SAR autofocus algorithms [8,9,10,11,12] are used to solve the problem in a blind mode. SAR autofocusing algorithms are categorized into three types: sub-aperture-based algorithms, prominent point-based algorithm, and metric-based autofocus. Most of the traditional autofocus algorithms assume there are strong scatters in the scene. Compared to the other conventional autofocus methods, the metric-based methods can work well without prominent points but deal with an already focused image.

The defocused image can be considered as the perfect focused image convoluted with the point spread function (PSF) caused by the phase error. Only recently and with the advent of lightweight and cheap SAR systems and UAV and drones, the problem of high cost of the system has pushed research to find new solutions in the development of such systems that imply in some cases the development of new techniques trying to focus the acquired raw data matrix using a subset of the ancillary data parameters and in presence of strong geometry anomalies that occur in such cases [13,14,15,16,17,18]. As the availability of the required parameters has always been guaranteed, at our knowledge, none has been done to develop algorithms able to estimate the reference function to be used as the focusing operator from the data itself and develop a completely blind focusing procedure.

While all the available algorithms to solve the SAR data-focusing problem are model driven, as they use ancillary parameters information to model the inverse problem in radar soil backscatter, in this paper a data-driven approach to develop a totally blind SAR data-focusing, based on the use of the Singular Values Decomposition (SVD) and LMS fitting of the phase information extracted from singular vectors is presented, able to obtain good image quality working on the complex SAR raw data matrix in absence of any information about the sensor. The proposed approach, at the state of the art, works well in the presence of a strong point scatterer in the scene.

The main idea is to exploit all the inherent information intrinsically stored in the data itself to extract the focusing reference functions to be used in a Ω-K or RD algorithm to obtain the Single Look Complex of any SAR sensor without even knowing important ancillary data parameters, needed by all the SAR focusing processors, such as the distance at the center of the beam, the radar sampling frequency, the transmitted chirp bandwidth, the chirp rate, and the chirp duration, the radar wavelength, the PRF, the sensor speed and the off nadir angle, used in data acquisition.

The proposed approach has been tested on several images of ERS raw data, made accessible for the scientific purpose from the Italian Space Agency (ASI) and the cross comparison with the state of the art focusing algorithm were carried out. The results indicate a good accordance to the standard focusing of obtained images with respect to the officially distributed ones. Also, the algorithm can reveal interesting and convenient in several application fields such as local zones monitoring by SAR systems carried by small lightweight and low-cost aerial unmanned vehicles. Modern hardware technology permits to reduce the size and weight of SAR systems into small and cheap flying platforms that can be conveniently used with low-cost platforms and flying drones. High-resolution microwave images of the observed scene can be obtained under various environmental conditions. Thus, UAV-SAR attracts growing interest in recent years [14]. The possibility of developing commercial low-cost systems is anyway still limited by the complication of the development of SAR due to the precise need for mission parameters to obtain good-quality images. Such parameters are very unstable for this kind of applications and the logging system introduces a complication, increasing their cost.

With respect to the reference [19] in which authors have preliminary proposed the blind technique to focus SAR data in the presence of a point scatterers in the scene, in this paper a more complete discussion about the quality of the focused image is carried out. To define the resolution in the range and azimuth directions, point scatterers responses are extracted from the image and azimuth and range cuts are compared with Range Doppler focusing of the same image. Also, an interferometric pair has been processed and the interference fringes have been extracted to show the good performance and phase stability of the proposed technique.

## 2. Materials and Methods

The proposed approach aims to show that the information needed to obtain the focused image can be extracted from the raw data itself when a point scatterer (either natural or manmade) is present in the scene. The problem is addressed with pattern recognition techniques.

In this section, the information exploitation method is presented. Initially the SVD approach used to extract the maximally correlated information inherently present in the image is introduced. The useful information of chirp in range and in azimuth are shown as extracted from the raw data. The SVD decomposition is discussed to explain the correlation exploitation mechanism. Once defined, the two reference functions extracted by the SVD, are used as inputs of a procedure needed to build clean versions, by LMS fitting procedure on the unwrapped phases both in range and azimuth. Clean versions of such signals are used to define the reference functions that are used to obtain the focused image. The reference functions are finally applied to obtain the focused image. To validate the algorithm, the proposed approach is then tested both on simulated data and on real data images. Focusing results have been carried out on several ERS1/2 raw data images showing the feasibility of the approach.

### 2.1. Blind SAR Data-Focusing Algorithm

The acquired data are the result of backscattering contribution of the ground at the SAR frequency. As a coherent illuminating source is used, the received data refer to several observations of the same scene taken in different points along the sensor platform trajectory. The radar transmitted pulses are stable in time, so the received returns show a strong azimuth correlation; several subsequent range lines in the raw data matrix contain roughly the same information so that the exploitation of coherence of the received signal can be attempted. This hypothesis allows the use correlation-based algorithm to extract useful information from data. The use of SVD technique can give us information about the reference functions to focus the image.

### 2.2. SVD—Signal Processing

SVD [20], in its economy formulation, is a standard algorithm able to decompose a given rectangular matrix into the product of three matrices, *U*, *S*, and *V*.

*X* is the data matrix of size M·N, *U*, and *V* are orthonormal matrices, respectively named the left and right singular vectors matrices; each singular vector, either left or right, is represented by the generic column of the matrix *U* or *V*, respectively. In analytic form, The SVD decomposition can be written simply as:

(1) $$X=U\cdot S\cdot V^{H}$$

where the superscript ${\left(\cdot \right)}^{H}$ represents the transpose and conjugate operator (Hilbert operator). *U* has the same size of the matrix *X* while *S* is a real valued diagonal matrix of order *N* and *V* is a complex orthonormal square matrix of order *N*:

(2) $$U^{H}\cdot U=I_{N}$$

and

(3) $$V^{H}\cdot V=I_{N}$$

*S* is the matrix containing the singular values of the matrix decomposition, sorted along the diagonal from the highest value to the lowest.

If a right multiplication for matrix V of both terms in (3) is applied, it can be usefully restated in another form:

(4) X·V=U·S=E

The *V* matrix is the matrix performing linear combinations of the columns of the data matrix *X* to obtain *E*, an orthogonal matrix. The right multiplication of the matrix *U* with *S* only scales the vector columns in *U* not affecting the orthogonality property.

As a simple example, the SVD decomposition of the matrix *X* made of only two columns requires the right singular vector matrix *V* an orthonormal matrix of size 2. The *V* matrix in this simple case represents a complex Gibbs rotation matrix. In this very simple case, the operation carried out by the decomposition becomes clear:

(5) $$\left[\begin{array}{cc} \;\vec{x_{1}} & \vec{x_{2}} \end{array}\right]\cdot \left[\begin{array}{cc} \;c & s\\ \;-s & c \end{array}\right]=\left[\begin{array}{cc} \;\vec{e_{1}} & \vec{e_{2}} \end{array}\right]$$

The *E* matrix is thus obtained as a simple linear combination of columns of *X*. *V* is the Gibbs rotation matrix and:

(6) $${\left|c\right|}^{2}+{\left|s\right|}^{2}=1$$

In a geometrical representation we can consider *c* and *s* parameters able to scale, rotate and phase shift vectors they multiply so that their sum and difference mixtures give the two orthogonal vectors in *E*. The modula of such vectors represent the singular values; it is easy to demonstrate that they are proportional to the estimates of the standard deviations of the resulting signals in *E*. When applied to a multicolumn matrix, this procedure tends to accumulate all the strongly correlated information on columns in the first left singular vector.

### 2.3. SAR Raw Data SVD Decomposition

When the *X* matrix to be decomposed is the raw SAR data matrix, the row index is the along track direction (azimuth) while the column index represents the slant range. The coherent illumination due to the transmission of the chirp produces very correlated information; in particular, in the azimuth direction the correlated information is the doppler phase history due to the scanning process. The expected result is that the first left singular vector should closely be related to the doppler history of the SAR system. As in *E* the transformed vectors are independent, the mostly correlated information in the signals in the columns of *X* is stored in the first singular vector in *E*, while the remaining part is in the others. Also, accordingly to the SVD decomposition scheme, the right singular vectors contain the mixing coefficients able to orthogonalize the raw data matrix.

This information is closely related to the transmitted information in the slant range direction. All the rows in the data matrix contain the same information, i.e., the transmitted chirp, delayed and phase shifted of an amount depending on the SAR geometry. The data are, again, strongly correlated, so the SVD decomposition will store in the rotation matrix *V* all the coefficients able to phase shift the subsequent transmitted chirps in a way that their sum convey the most important part of the global transmitted energy of the pulse. The coefficient must then be closely related to both the geometrical properties of the SAR acquisition and the characteristics of the transmitted chirp. This consideration will be illustrated in the next paragraph. Basing on these observations, a simple and direct scheme to obtain a good focusing of the raw data in a blind mode has been devised.

## 3. Discussion

### 3.1. B-SAR—Blind SAR Data-Focusing Algorithm

**Figure 2:**
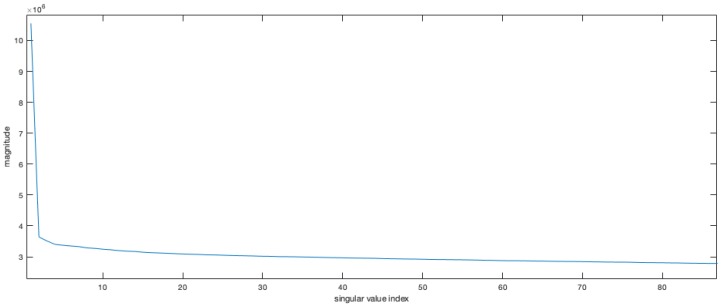
Singular values for the SAR raw data matrix decomposition. On abscissa the indexes of the singular values, on the vertical axis the magnitude. The first singular value is very high compared to the others showing that a large correlation was present in the raw data matrix and exploited in the first left and first right singular vectors.

The focusing algorithm acts as a simple SVD decomposition. Once obtained, the first left and first right singular vectors can be used to define properly the reference functions to be used in the focusing procedure. Figure 2 shows the plot of the elements of the diagonal matrix *S*. As the matrix *E* contains orthogonal components, the energy of the whole image can be computed as the sum of the squares of the singular values. In the matrix a large part of the energy remains confined in the first left and first right singular vectors of matrices *U* and *V*.

**Figure 3:**
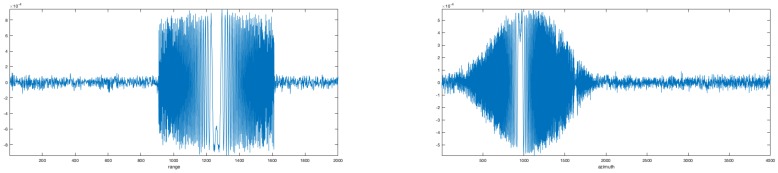
Real parts of first right and first left singular vectors for the SAR raw data matrix decomposition. The range amplitude results almost constant, as the transmitted chirp is assumed to be. The azimuth singular vector is shaped by the azimuth antenna beam pattern.

An example of the first right and first left singular vectors of the SVD decomposition are shown in Figure 3.

From the simple observation of the first left singular vector of the matrix *U* two parameters can be extracted: the antenna beam pattern in azimuth and the phase history of the azimuth reference to be used in the focusing procedure. To select the proper parts of the two singular vectors for the focusing procedure a threshold was selected aiming at retaining the portion of the signal with amplitude greater than a given percentage of its peak value. In this paper, the 10% was selected. This allows to define the reference functions both in range (working on the right singular vectors) and in azimuth (left singular vectors).

In real cases, a noisy appearance of the two signals is expected (see Figure 3); for this reason, instead of using the two so extracted reference functions in the focusing procedure, the unwrapped phases of such functions were extracted to define their clean versions and reduce the noise effects on the focused image.

Starting from the extreme points of such estimated phase functions a LMS polynomial fitting was used to construct a clean version of the phase histories to be used in the description of the two reference functions, both in azimuth and in range. The unwrapped phase of the reference function in either range or azimuth was approximated by a polynomial function and used to clean up the phase of the reference functions.

Stated *y* the polynomial function to be used to approximate the unwrapped phase of the reference function, the set of coefficients was computed via a LMS fitting procedure.

(7) $$y=\sum_{i=1}^{N}a_{i}\cdot x^{i}$$

Here, $a_{i}$ represent the polynomial coefficients and *x* the index of samples of the reference function.

**Figure 4:**
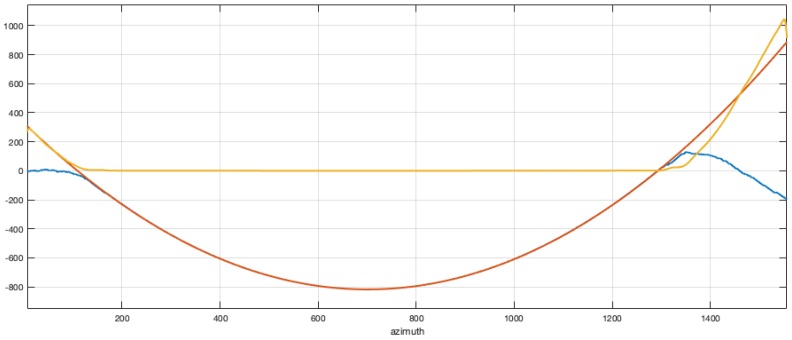
Phase as extracted from the first left singular vector (blue), the phase polynomial LMS approximation (red) and the phase error (yellow).

Figure 4 represents the three diagrams of the unwrapped phase of the reference function, the polynomial approximation, and the phase error.

The reference function unwrapped phase estimated with the described procedure was selected in an interval of values in which the phase difference between subsequent samples is not larger than π. This choice allows to contain the aliasing effect due to the sampled phase history. To further reduce the aliasing effect at borders of the reference functions, raised cosine tunable length windowing was used.

### 3.2. Experimental Results

In this section, a simulated experiment of a point target in Additive White Gaussian Noise (AWGN) is presented to show the results that can be obtained using the SVD decomposition; here, all the information inherently present in the raw data file is exploited; in the successive subsections, results of the focusing procedure are presented for real SAR raw data.

#### 3.2.1. Simulated Experiment

To test the performance of the proposed algorithm a simulated point target on a noise floor has been used. The simulated azimuth antenna beam pattern was shaped by a Hanning window. The simulated raw data matrix was affected by AWGN with a low SNR. The ERS mission parameters are used in the simulation and reported in the Table 1.

The obtained raw data matrix was decomposed using the SVD algorithm. A large part of the energy in the data matrix is concentrated in the first singular value, clearly stating that the first left singular vector (i.e., the first column of matrix *U*) should contain the orthogonal signal with maximum energy in the data.

To select the proper length of the range chirp history and reconstruct a clean reference in range, a thresholding procedure was used; after the SVD decomposition, the reference signal power is much higher than the background noise, so this procedure reveals effective. In this case, the selected range time duration by the choice of a threshold at 10% of the signal peak value was efficient. After the procedure described in the previous subsection, a clean range reference function was obtained.

The same procedure was carried out for the azimuth reference, with a slight more care: the antenna beam pattern estimated in this way is not always effective due to the growing attenuation and the joint influence of azimuth and range beam patterns with the slant range and the possible presence of wide strong scatterers that can reduce the quality of the estimated pattern. Also, it is not clear, in this case, where the azimuth phase history should be stopped. The main objective is to limit the phase history in a proper way to avoid azimuth aliasing. The phase history was selected in a generally asymmetric interval around the peak of the unwrapped phase of the reference function: the possible presence of a squint angle in the true acquired data forces to select the proper doppler phase history to cover all the useful part of the extracted signal phase. As for the range, the thresholding procedure used in the azimuth reference definition was limited to the interval in which the signal was above the 10% of the estimated peak of the beam pattern. Once extracted, anyway, the selection of the proper portion of the phase history to be used in the construction of a clean reference was made basing on the phase, as discussed previously (cfr. Figure 4). Tunable length tapered tails were used to control aliasing effects without reducing severely the azimuth resolution of the focused image, both in the range and azimuth reference functions.

**Figure 5:**
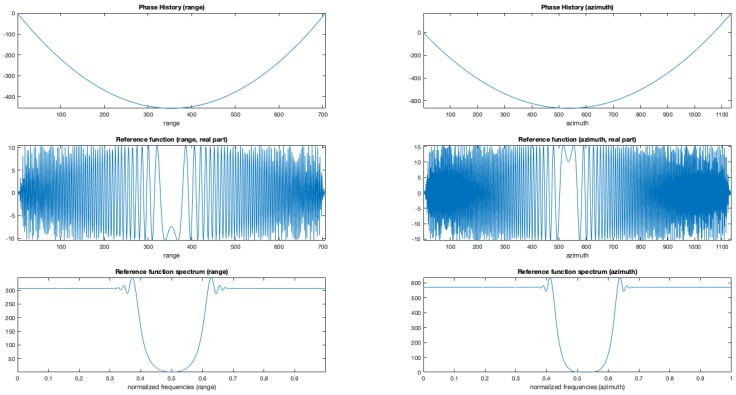
Range and Azimuth references to be used in the focusing procedure. Left range, right azimuth. The three rows present extracted phase histories, the real parts of the reference functions and their spectra.

Figure 5 reports the obtained reference functions used to focus on range and azimuth the raw data. Figure reports the range (left column) and azimuth (right column). On the rows, the phase, the real part, and the spectrum of each reference function is presented.

**Figure 6:**
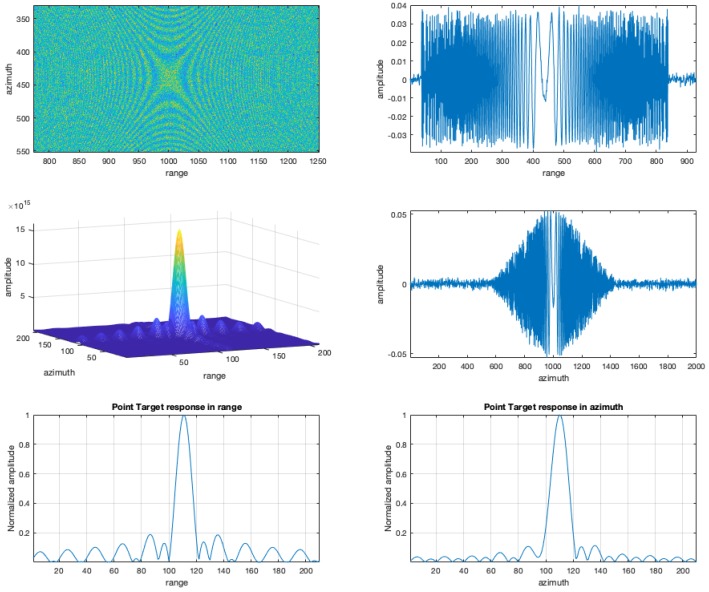
Results of simulated experiment. Top left: real part of the simulated (with additive gaussian noise) raw data; top right: First left singular vector as extracted from the SVD decomposition. center left: The 3D version of the (× 10 interpolated) focused pulse detail of the simulated raw data with the B-SAR algorithm; center right: First right singular vector as extracted from the SVD decomposition. It refers to the azimuth direction, showing a (hamming simulated) shape of the antenna beam pattern; bottom left: the range cut of the interpolated focused pulse; bottom right: the azimuth cut of the interpolated focused pulse.

Figure 6 reports the obtained focusing results for the simulated case. Specifically, the figure shows the real part of the simulated (with additive gaussian noise) raw data the first left and right singular vectors as extracted from the SVD decomposition, the 3D version of the (× 10 interpolated) pulse of the simulated raw data focused with the B-SAR algorithm, the two cuts, in range and azimuth of the interpolated focused pulse. The proposed approach is thus simple and direct and allows to extract useful information to focus the received data.

It should be pointed out that the possibility of obtaining good estimates of the range and azimuth chirp responses is due to the clear presence of a point scatterer with a sufficiently high signal to noise ratio that conveys a large part of the data matrix energy.

Once the azimuth and range reference functions have been defined, focusing can become simple and can be carried out with either RD or Ω-K algorithms. In this paper, the frequency approach has been used to obtain the focused image.

**Figure 7:**
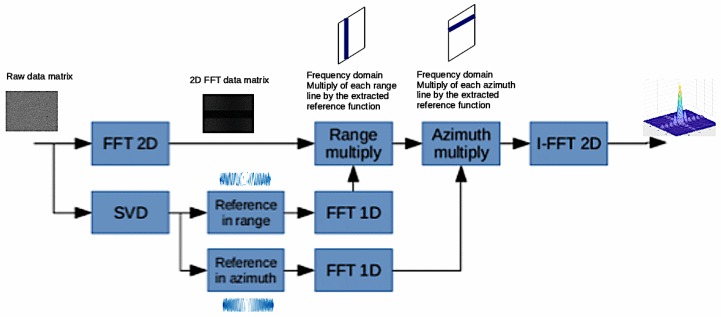
B-SAR Data Processing block diagram.

A block diagram of the complete algorithm is reported in Figure 7.

#### 3.2.2. Real SAR Raw Data-Focusing

The SVD decomposition was then applied as a test of the proposed approach to several ERS raw data matrices without any knowledge of the mission ancillary data.

**Figure 8:**
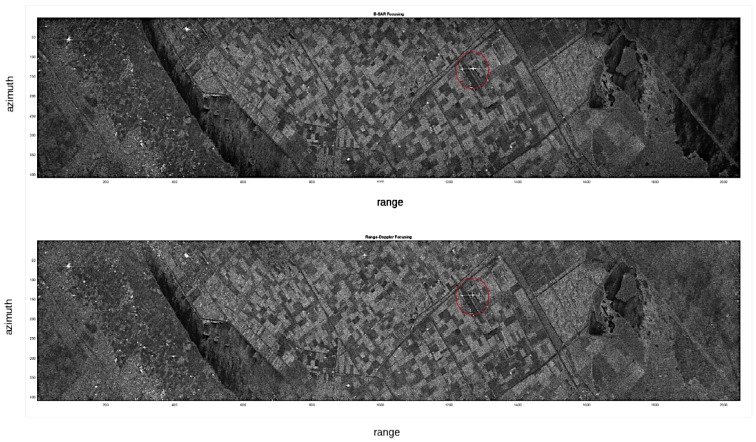
Sample of focused image with the proposed algorithm (upper) compared with the standard focusing obtained via Range Doppler algorithm (lower). ERS 1—Matera. The red circle indicates the point scatterer.

The thresholding applied on the range reference function estimated a length of the range chirp of 704 samples. In Figure 8, the focused ERS image obtained with the proposed algorithm (B-SAR) and the focusing obtained by a standard Range Doppler SAR processor are compared.

**Figure 9:**
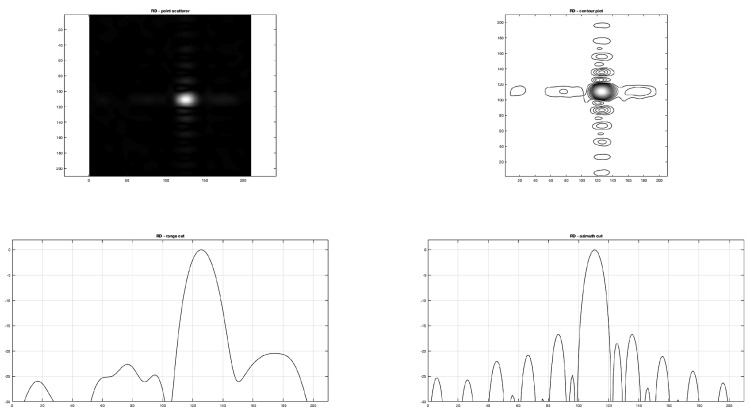
Range Doppler algorithm. Point scatterer image, contour plot, and range and azimuth cuts.

**Figure 10:**
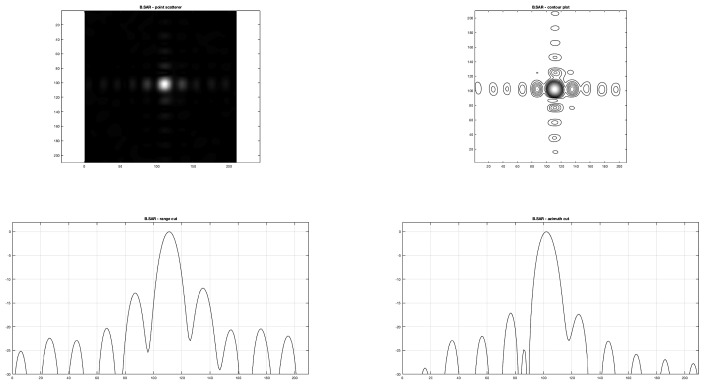
B-SAR algorithm. Point scatterer image, contour plot, and range and azimuth cuts.

The better appearance on the focused image is due to the normalization process. The images have been normalized with the same algorithm that amplifies the focused revealed image to a fixed value after normalization to the standard deviation of the whole image. The appearance of the image brightness is different because of the circular convolution in the Range Doppler procedure due to the frequency domain processing (its effect can be noted on the horizontal axis due to the presence of a periodic structure in the image that folds around the image). No zero padding was used in this procedure in the Range Doppler focusing software, while in the proposed algorithm the zero padding was used both in range and azimuth directions to avoid the circular convolution distortion. This reduces the amplitudes of large portions of the image, giving rise to a lower standard deviation and in the normalization a higher factor. This higher factor enhances very much the point scatterer that appears larger on the image, but the range and azimuth cuts in Figure 9 and Figure 10 allow a better comparison of the performances.

### 3.3. Semi-Quantitative Evaluation of Focusing Performance

In this paragraph the comparison between the range and azimuth cuts of the proposed B-SAR and RD algorithm is carried out.

In particular, in Figure 9 and Figure 10 the range and azimuth cuts and the contour shaping of the same point scatterer on respective images obtained focusing the raw SAR data matrix are presented, showing how the range focusing obtained by the proposed procedure seems to adhere more precisely to the theoretical one than the RD focusing.

On the other side, at a lower resolution achieved by RD in the range direction, a higher rejection of side lobes is obtained, showing that the clean aspect of the focused image is due to a higher smoothing of range lines.

**Figure 11:**
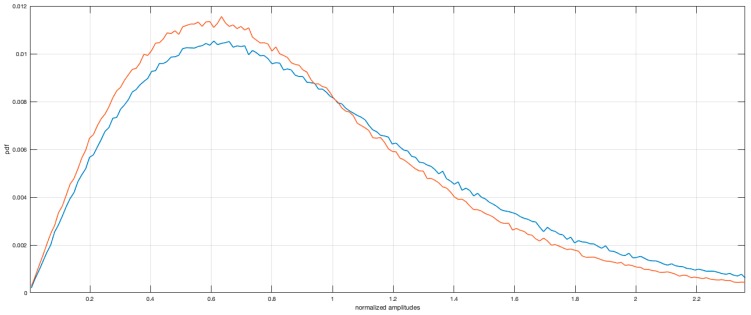
Histogram of focused images amplitudes comparison.

For the two focused images, a comparison of the amplitude statistics for the images focused with standard RD approach and B-SAR are plotted in Figure 11. What is evident is a close adherence of the statistics of the B-SAR image to the RD one, showing anyway a more compactness in the values, symptom of an imperfect focusing of highest peaks for this approximated method. The possibility of using the proposed algorithm in several applications in the field of Earth observation, interferometry and multi-temporal interferometry should be a goal to pursue even when mission parameters are unknown. To assess the performance obtained by the proposed approach with respect to the specific parameters of the ERS missions, a comparison of some specific radiometric parameters was carried out. The radiometric parameters table for ERS mission is reported in the Table 2.

Using these parameters, the theoretical number of samples of the transmitted chirp can be computed as the product of the ERS sampling frequency and the chirp duration, with a close adherence with our estimate of 704 samples of the proposed technique. Also, the theoretical relative bandwidth of the chirp can be obtained as the ratio between the chirp bandwidth and the sampling frequency. For our algorithm, the estimate of this parameter can be computed as the equivalent bandwidth of the reference in range function. Also, in this case a close adherence of the theoretical and experimental relative bandwidth has been obtained, as reported in the Table 3.

#### 3.3.1. B-SAR Phase Stability

**Figure 12:**
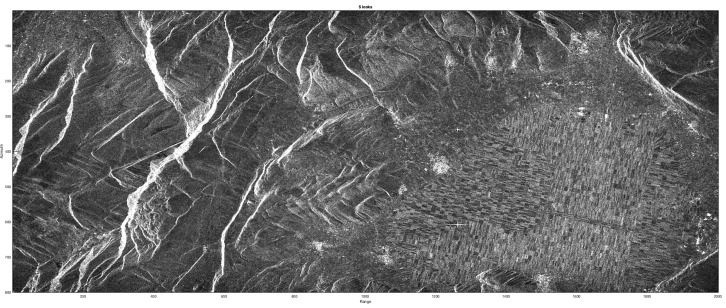
B-SAR focusing. Caramanico site, Fucino Valley, Italy.

**Figure 13:**
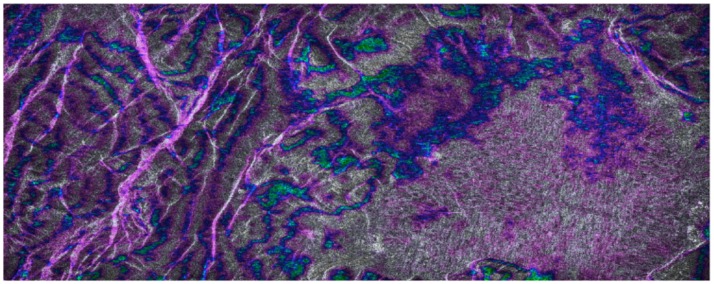
B-SAR, 5 looks, slope corrected interferometric image obtained by B.SAR focusing algorithm with superimposed the intensity image. Caramanico site, Fucino Valley, Italy.

An interesting parameter for the proposed approach for blind SAR data-focusing is its phase stability: the interferometric image has been computed on real ERS1/2 tandem pair pass over the Fucino region in Italy. Figure 12 shows one of the focused images of the tandem pass pair, while Figure 13 shows the (5 looks, slope corrected) interferometric image obtained by B-SAR focusing algorithm with superimposed the intensity image. A close correspondence between the flat zones in the valley with the smooth variations of the phase seem to assess the good behavior and the phase stability of the proposed processing technique.

#### 3.3.2. Processing Time

The description of the time processing required by the proposed approach to obtain the focused image is reported in Table 4 with respect to the original size of the raw data matrices. The experiments have been conducted on a desktop pc equipped by an Intel Core i7 Processor with clock speed of 3.4 GHz and a total number of four cores, and a memory of 16 GB.

The processing times needed to obtain the SLC/PRI images reported in the table are comparable to the standard processing times of other algorithms.

## 4. Conclusions

In this work the SVD decomposition has been used to extract correlated information from SAR raw data on scenes where a strong point scatterer is present. The use of the SVD is a sufficient information allowing the development of a simple and direct procedure to focus the acquired data without the need for information about the sensor attitudes, path, and SAR system parameters. The aim of this paper is to define a simple and direct method to obtain good focused images for several application, such as aerial archaeology inspection, agriculture, change detection for land usage and so on. The availability of a blind focusing algorithm can allow the development of simpler SAR systems to be used in low-cost applications in which the highest precision in the focused image is not a strict requirement.

To deal with the problem of precise focusing of the entire image taking care the range space variant system impulse response can be addressed, in a post processing way, using one of the several available autofocusing techniques available in the scientific literature.

The proposed algorithm, at the state of the art, is sufficient to obtain a fair focused image. The appearance of the focused image obtained is comparable with standard RD focusing, as shown in Figure 9. To assess the performance of the proposed approach, point scatterer responses have been compared between the RD and B-SAR focused images, showing a pretty good correspondence.

Also, the problem of phase stability of the algorithm has been addressed, computing the interference fringes corresponding to an ERS 1-2 tandem mission, showing also good adherence of the specific local orography. The main limitations of the proposed algorithm depend on its need for the presence of a strong point scatterer in the imaged zone. This limitation is payed back by its simplicity and the lack of need for the ancillary parameters file in the focusing procedure, aspect that simplifies both the processing and the development of simple and cheap SAR systems to be used in local monitoring also with the recourse to simple aerial unmanned vehicles such as drones.

The focused image is obtained, at the state of the art, by SVD analysis. This algorithm performs correlation exploitation of the contributes of the several azimuth lines. This leaves some room for further analysis as the residual correlation is inherently present in the lower singular vectors and not only on the first one, meaning that better image quality can be addressed using all the correlated components in the SVD decomposition. Of course, the problem is crucial, and attention is being taken on this subject. A precise focusing algorithm taking care of the Range Cells Migration Compensation is the goal of our future work. Recently, some authors have presented good results for SAR With Nonlinear FM Chirp Waveforms [21]. This specific case has not been addressed in this paper and will be the goal for future research. The computational aspects to obtain good-quality focused images are also important: recently some studies about efficiency have been presented [22] exploiting the multicore-based architectures of modern processors. Also, this aspect needs further research, as the proposed approach pays the cost of no information available for the SAR sensor with an increase of computational complexity. Also, the possibility of blind focusing SAR raw data, here addressed only in the presence of a point scatterer (e.g., a corner reflector or a transponder), in the general case of SAR strip map data-focusing represents the field of application for future work.

The proposed algorithm, developed in MATLAB, is distributed under the Noncommercial—Share Alike 4.0—International Creative Common license by the authors.

## Figures and Tables

**Table 1:** Parameters used to simulate raw SAR data.

	Parameters	Units
Carrier Frequency	$f_{c}=5.300$	GHz
Chirp Duration	$T_{c}=37.12$	μs
Chirp Bandwidth	$B_{ch}=15.50829$	MHz
Sampling Frequency	$f_{s}=18.962$	MHz
Satellite height	h=700	km
Satellite speed	$v_{s}at=11.75$	km/s
Off Nadir Angle	θ=23	deg
Squint Angle	ϕ=0	deg

**Table 2:** ERS radiometric parameters.

	Parameter	Units
Carrier Frequency	$f_{c}=5.300$	GHz
Chirp Duration	$T_{c}=37.12$	μs
Chirp Bandwidth	$B_{ch}=15.50829$	MHz
Sampling Frequency	$f_{s}=18.962$	MHz

**Table 3:** Comparison of Theoretical and Estimated Chirp Relative Bandwidth.

	Value
Theoretical Relative Bandwidth	0.8178
Estimate of the Relative Bandwidth	0.8164

**Table 4:** Processing times.

Image Name	[Rows, Cols] (Complex)	Proc. Time [s]
Caramanico 1	[2001, 4001]	21.049431
Caramanico 2	[2001, 4001]	20.528043
Flevoland	[1101, 5000]	13.358860
Matera	[2048, 2048]	10.970114
Simulated Pulse	[776, 2001]	2.473084
